# Evaluation of Aflibercept Treatment Responses in Eyes with Bevacizumab/Ranibizumab-resistant Wet Age-related Macular Degeneration

**DOI:** 10.4274/tjo.34735

**Published:** 2017-06-01

**Authors:** Tuncay Topal, Taner Kar, Yıldıray Yıldırım, Sercan Koray Sağdıç, Cihan Büyükavşar, Abdullah Kaya, Ali Ayata, Murat Sönmez, Melih Hamdi Ünal

**Affiliations:** 1 Haydarpaşa Sultan Abdülhamid Training and Research Hospital, Ophthalmology Clinic, İstanbul, Turkey; 2 Anıttepe Military Dispensary, Ankara, Turkey

**Keywords:** Aflibercept, central retinal thickness, visual acuity, pigment epithelial detachment

## Abstract

**Objectives:**

To evaluate anatomic and functional results after switching from intravitreal bevacizumab or ranibizumab treatment to aflibercept for wet (neovascular) age-related macular degeneration.

**Materials and Methods:**

This retrospective study included 22 eyes of 22 patients resistant to treatment with at least 6 injections of bevacizumab or ranibizumab. The first three injections had been applied monthly, the others pro re nata (PRN). Outcome measures were follow-up period, injection number, best corrected visual acuity (BCVA), central retinal thickness (CRT) and pigment epithelial detachment (PED) height. Dosing regimen of aflibercept was determined PRN. The patients were examined monthly. In all visits, BCVA and optical coherence tomography results were assessed together and injections were applied according to these findings. Patients with at least three months of follow-up were included in the study.

**Results:**

Twenty-two eyes of 22 patients treated with bevacizumab or ranibizumab were switched to aflibercept therapy. Seven patients had serous PED and 4 patients had fibrovascular PED. The mean follow-up periods for these groups were 20.59±6.76 months and 8.68±3.79 months, respectively. The mean injection numbers were 10.5±3.61 vs 4.54±1.56. Statistically significant reductions were noted in CRT (533.86±164.06 µm vs 412.04±143.86 µm, p<0.05). BCVA levels were almost equal before and after switching (0.18±0.17 vs 0.18±0.14). Serous and fibrovascular PED heights decreased suboptimally from 460±281.51 µm to 282.42±175.76 µm (p>0.05) for serous PEDs and 251.25±43.85 µm to 225.75±73.09 µm (p>0.05) for fibrovascular PEDs.

**Conclusion:**

Switching to aflibercept resulted in significant improvement in CRT, but not in BCVA or PED heights.

## INTRODUCTION

Age-related macular degeneration (AMD) is the foremost cause of severe vision loss, particularly in populations over 55 years old in developed countries. The prevalence of AMD in individuals 40 years and older is estimated as 6.5%.^1^ The condition is a chronic, degenerative process and is divided into the non-neovascular atrophic (dry) type and the neovascular (wet) type. Approximately 10-20% of all AMD patients exhibit the wet type, which is responsible for about 80% of vision loss due to its rapidly progressive and destructive course. The characteristic feature of wet AMD is neovascularization that originates from the choroidal vasculature and extends to the subretinal pigment epithelium or subretinal space. Though overexpression of the proangiogenic cytokine vascular endothelial growth factor (VEGF) has been shown to be the main cause, the pathogenesis of choroidal neovascularization (CNV) has not been fully elucidated. VEGF released by astrocytes and Müller cells due to ischemia and other secondary factors triggers the neovascular process by stimulating endothelial cell proliferation and migration. These neovascular structures cause hemorrhage, fluid accumulation, or fibrovascular tissue, which disrupt the retinal and subretinal anatomy, resulting in vision loss.

Laser photocoagulation and photodynamic therapy (PDT) were used to treat wet AMD from the 1980s until the early 2000s. As the importance of VEGF’s role in the pathogenesis of wet AMD became better understood, anti-VEGF agents were favored over these treatment modalities. 

Bevacizumab and ranibizumab, the agents most commonly used in the management of wet AMD, inhibit all isoforms of VEGF-A. Aflibercept binds VEGF more strongly, for a longer time and with higher affinity than bevacizumab, ranibizumab or the body’s VEGF receptors, and shows lower antigenicity.^2,3^ Unlike the other two anti-VEGF agents, aflibercept also inhibits VEGF-B and platelet-derived growth factor (PDGF) and is able to penetrate through all the retinal layers and under the retinal pigment epithelium. The half-life of aflibercept (7.13 days) is shorter than that of bevacizumab (8.25 days) and longer than that of ranibizumab (4.75 days).

Wet AMD patients under long-term treatment with bevacizumab or ranibizumab may exhibit persistent subretinal fluid and exudative changes. For such patients who are resistant to these agents or show only partial or suboptimal response, it has been posited that changing their intravitreal treatment to aflibercept may be an effective approach.

The purpose of this study was to evaluate the functional and anatomic outcomes of intravitreal aflibercept injection in patients with wet AMD refractory to intravitreal bevacizumab or ranibizumab therapy.

## MATERIALS AND METHODS

This retrospective study included the medical records of wet AMD patients who had intraretinal and/or subretinal fluid resistant to at least 6 intravitreal bevacizumab or ranibizumab injections and were switched to aflibercept therapy between January 2014 and August 2015. The study was approved by the Ethics Committee Chair of the Haydarpaşa Numune Training and Research Hospital. Informed consent forms were obtained from all patients prior to injections. Optical coherence tomography (OCT) images were obtained using a spectral OCT-Scanning Laser Ophthalmoscope (Spectral OCT-SLO, Optos, Scotland).

Prior to changing therapeutic agents, the patients received one injection of a loading dose of bevacizumab or ranibizumab per month for the first three months; thereafter, injections were performed as deemed necessary based on a combination of OCT and best corrected visual acuity (BCVA) values obtained during monthly follow-up examinations. After the first three loading doses, repeat injections were applied if monthly follow-up revealed more than one line loss in BCVA or an increase of more than 100 µm in central retinal thickness (CRT). Patients with persistent intraretinal and/or subretinal fluid on OCT, no improvement in BCVA, or a CRT increase of more than 100 µm compared to baseline after at least 6 injections were considered resistant to bevacizumab/ranibizumab therapy and upon obtaining consent it was decided to change to intravitreal aflibercept therapy. Patients were examined for any possible complications of intravitreal injection on the first day after treatment and were followed monthly thereafter. The same criteria for repeated injections of the other agents were applied for aflibercept in patients’ monthly follow-up examinations. The study included patients who were followed in this manner for at least three months. Changes in final BCVA, CRT and pigment epithelial detachment (PED) values on OCT from baseline were recorded and compared with pre-injection values.

Inclusion criteria of the study were: 1) age 50 years or older and wet AMD diagnosis; 2) treated with at least 6 intravitreal bevacizumab or ranibizumab injections; 3) followed in our outpatient clinic for at least 3 months after switching to aflibercept therapy; and 4) absence of any disease other than wet AMD that may cause macular edema or atrophy. Exclusion criteria were: 1) history of ocular procedures other than uncomplicated cataract surgery or Nd:YAG laser posterior capsulotomy; 2) any history of PDT; 3) any ocular or systemic conditions other than wet AMD which may cause macular edema or CNV.

All injections were performed in operating room conditions. Injections were performed with 30 gauge needles and doses of 1.25 mg bevacizumab, 0.5 mg ranibizumab or 2 mg aflibercept in a volume of 0.05 mL, injected intravitreally 3.5 mm from the limbus in pseudophakic eyes and 4 mm in phakic eyes.

### Statistical Analysis

Statistical Package for the Social Sciences (SPSS) version 17.0 software was used for all statistical analyses. Descriptive statistics are expressed as minimum, maximum and mean ± standard deviation. The Wilcoxon test was used for paired samples. P values <0.05 were accepted as statistically significant.

## RESULTS

A total of 22 eyes of 22 patients with wet AMD received aflibercept injections. The patients’ ages ranged from 50 to 90, with a mean of 74.9±9.92 years. Ten patients were male, 12 were female. Nine eyes were right, 11 were left. PED was present in 11 patients when therapy was switched to aflibercept, 7 with serous PED and 4 with fibrovascular PED. The mean number of intravitreal bevacizumab or ranibizumab injections was 10.5±3.61 (range, 6-21) and the mean number of aflibercept injections was 4.54±1.56 (range, 2-8). The mean follow-up time was 20.59±6.76 (range, 8-36) months before switching to aflibercept and 8.68±3.79 (range, 3-15) months after switching to aflibercept. CRT was 533.86±164.06 (range, 300-890 µm) at first aflibercept injection and 412.04±143.86 µm (range, 171-712 µm) at the end of follow-up; this difference was statistically significant (p=0.024). Serous PEDs had a mean height of 460±281.51 µm (range, 185-975 µm) at baseline and 282.42±175.76 µm (range, 59-519 µm) at final examination. Fibrovascular PEDs had a mean height of 251.25±43.85 µm (range, 159-318 µm) at baseline and 225.75±73.09 µm (range, 176-320 µm) at final examination. Neither PED type showed a statistically significant reduction in height after aflibercept injections (p=0.12 and p=0.71, respectively). Mean BCVA values were 0.18±0.17 prior to first aflibercept injection and 0.18±0.14 at the end of follow-up (p=0.51). The findings of this study are summarized in Table 1.

No complications due to intravitreal injections were observed during follow-up.

## DISCUSSION

The majority of wet AMD patients require repeated intravitreal injections in the long term. The need for long-term monthly injections may be related to the pathologic activity becoming chronic, but may also arise due to drug tachyphylaxis, tolerance development, or an immune reaction to a component of the injected solution. In tachyphylaxis, there is no response to treatment, even at higher drug concentrations resulting from frequent repeated drug administration. However, efficacy may return if the medication is discontinued for a period of time. Tolerance is also a significant reduction in the extent and duration of a drug’s efficacy as a result of long-term application. In such cases, efficacy can be increased by reducing the dosage or intervals between applications.^4^ Unlike tachyphylaxis, discontinuing treatment after the development of drug tolerance does not restore efficacy. Gasperini et al.^5^ reported that 81% of patients with bevacizumab or ranibizumab tachyphylaxis showed improved response after switching therapies. Local or systemic immune responses after intravitreal injections may arise due to the development of antibodies against one of the injected substances. Several authors have proposed that chronic VEGF blockage leads to overexpression of VEGF by macrophages in choroidal neovascular tissue.^6,7,8,9^

Aflibercept is now used for wet AMD patients resistant to the other anti-VEGF agents. The molecular structure of aflibercept results in a binding affinity 94 times greater than that of bevacizumab and 119 times that of ranibizumab. Aflibercept also inhibits other angiogenetic agents such as VEGF-B and PDGF.^10,11,12,13^ The intraocular duration of effect of aflibercept is 48-80 days.^14^

There are studies documenting the efficacy of aflibercept in refractory wet AMD in terms of anatomic rather than functional success.^15,16,17,18^ One such study by Yonekawa et al.^16^ evaluated BCVA and CRT in 102 eyes of 96 patients who were switched to aflibercept after developing resistance to ranibizumab; their results showed that BCVA remained stable while CRT was significantly reduced. CRT decreased significantly in 91% of the patients and remained unchanged in 9%; no cases of increased CRT were observed. In contrast to this study, others have reported improved visual acuity after aflibercept therapy. Heussen et al.^17^ evaluated 71 eyes of 65 patients with refractory wet AMD who were switched to aflibercept and reported a 33% increase in BCVA.

**Figure 1:**
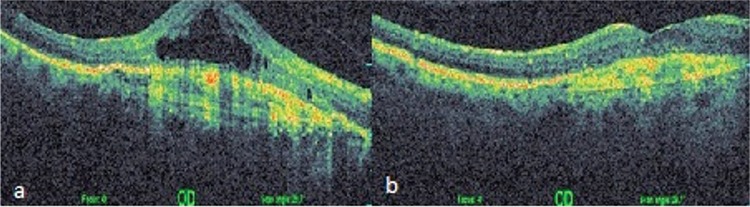
a) Before aflibercept therapy, central retinal thickness was 650 µm and intraretinal fluid and cysts were apparent, b) 15 months after 8 aflibercept injections, central retinal thickness was 347 µM, intraretinal cyst had resolved, and fluid was reduced. The patient’s best corrected visual acuity increased from 0.12 to 0.3

**Figure 2:**
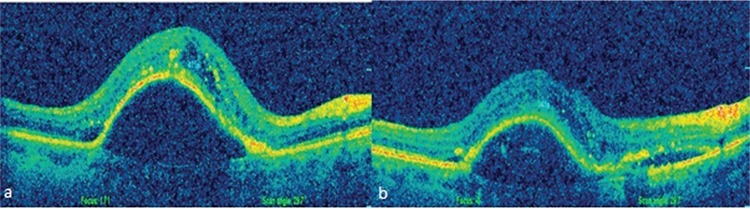
a) Before switching to aflibercept therapy, pigment epithelial detachment height was 975 µm, best corrected visual acuity was 0.4, b) 8 months after 5 injections, central retinal thickness was 345 µm, pigment epithelial detachment height was 519 µm, best corrected visual acuity was 0.3

In the present study, we observed no significant increase or decrease in BCVA but found a significant reduction in CRT. According to OCT findings, intraretinal or subretinal fluid completely resolved in 6 of 22 eyes (Figure 1a, 1b), partially resolved in 9 patients, remained nearly the same in 2 patients (Figure 2a, 2b), and increased in 5 patients following aflibercept injection. We believe long-term retinal damage due to persistent intraretinal and/or subretinal fluid prevented significant improvement in BCVA. The patients in our study had persistent intraretinal and/or subfoveal fluid despite an average of 10.5 intravitreal injections prior to switching to aflibercept. Though the inability of aflibercept to effect significant visual improvement may be attributable to advanced photoreceptor damage resulting from chronic fluid accumulation prior to treatment, it may also be related to not administering the loading dose of aflibercept.

**Figure 3:**
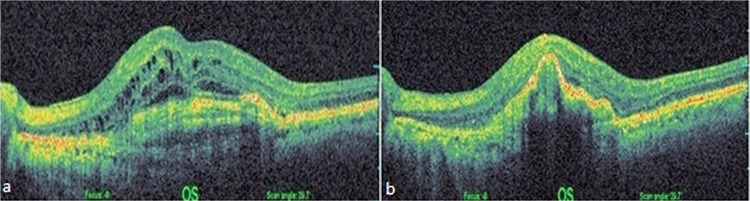
a) Before switching to aflibercept therapy, central retinal thickness was 695 µm and intraretinal cysts and fluid were observed. There was fibrovascular pigment epithelial detachment and best corrected visual acuity was 0.2, b) 8 months after 3 injections, central retinal thickness was 300 µm and the cysts and fluid were nearly completely resolved, but pigment epithelial detachment height increased and best corrected visual acuity was still 0.2

Many studies have investigated the relationship between PED type and anti-VEGF treatment response. Hoerster et al.^18^ reported that fibrovascular PED is resistant to ranibizumab therapy, while serous PED responds well. Inoue et al.^19^ observed reduced height in 100% of serous and mixed-type PEDs versus 67% of fibrovascular PEDs. In the present study, both serous and fibrovascular PEDs decreased in height, but these reductions were not statistically significant (Figures 2a, 2b and 3a, 3b).

Various administration protocols for aflibercept have been documented in the literature. Horizon AMD, Secure and Seven-up studies reported that the best visual acuity and anatomic results are achieved with monthly regimens.^20^ García-Layana et al.^21^ reported comparable results from ranibizumab applied monthly and aflibercept applied once every two months. Batioglu et al.^22^ administered 3 loading doses of aflibercept followed by repeated injections as needed based on examination and OCT findings, and reported no significant increase in BCVA but significant decrease in CRT in their patients. In the present study, we administered repeated aflibercept injections as needed based on BCVA and OCT findings after one initial injection and achieved only anatomic success.

### Study Limitations

Limitations of our study are the retrospective method, the small patient number and administering aflibercept according to the pro re nata protocol, without giving the three-part loading dose.

## CONCLUSION

To summarize, switching patients with refractory wet AMD to aflibercept therapy resulted in significant CRT reduction, but significant changes were not achieved in terms of BCVA improvement or decrease in PED height. The role of aflibercept in the management of wet AMD may be better clarified by future studies with larger patient numbers, longer follow-up times, and patients who were initially treated with aflibercept after being diagnosed with wet AMD.

## Figures and Tables

**Table 1:**
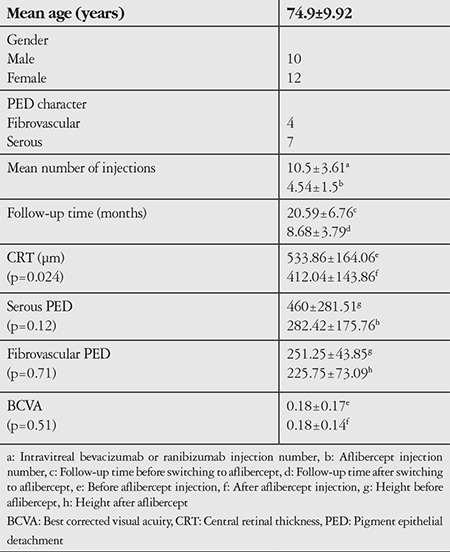
Patients’ demographic and clinical characteristics
